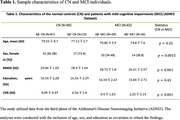# Distinct associations between the neurite microstructure of white matter bundles, tau pathology and cognitive decline in preclinical Alzheimer's disease

**DOI:** 10.1002/alz.093222

**Published:** 2025-01-09

**Authors:** Elveda Gozdas, Hadi Hosseini

**Affiliations:** ^1^ Stanford University, Stanford, CA USA

## Abstract

**Background:**

Recent advancements in molecular positron emission tomography (PET) enable precise tracking of tau pathology in Alzheimer's disease (AD). Tau pathology typically begins focally in the medial temporal lobe, rapidly expanding due to amyloid‐β (Aβ) influence. This expansion may lead to neurodegeneration along connected pathways to the tau epicenters, resulting in cognitive decline. Our study utilized PET scans, diffusion Orientation Dispersion and Density Imaging (NODDI), and longitudinal memory data to investigate the intricate connections between tau accumulation and neurite microstructures in white matter bundles connected to the tau epicenter and resulting cognitive decline.

**Method:**

The study had 155 participants, including 90 cognitively normal (CN) and 65 mild cognitive impairment (MCI) from the Alzheimer's Disease Neuroimaging Initiative dataset (Table 1). The study included cortical tau accumulations in the entorhinal cortex (EC) to the hippocampus (H) and neurite microstructures of the cingulum hippocampal bundle (CHB). The study also included longitudinal episodic memory scores measured using the Rey Auditory Verbal Learning Test Immediate (RAVLT‐I).

**Result:**

The association between tau accumulation in EC and H in the right and left hemispheres was found to be significantly related to the increased isotropic water (FISO) of HCB (p<0.01). In contrast, the fiber complexity (ODI) along the CHBshowed a correlation with tau accumulation in the right EC (p=0.03, t=2.31) and H (p=0.02, t=‐2.33) for the entire sample. It's worth noting that significant correlations between FISO and ODI and tau accumulations in the epicenters remained significant for the amyloid‐positive group (Aβ^+^ CN and MCI). Also, the tau aggregation in the retrosplenial cortex (RSC) was significantly correlated with the neurite layout of CHB (p<0.05). Finally, we explored the correlation between the microstructural changes in neurites along the CHB and cognitive decline. The linear mixed effect model showed a significant interaction effect of tau_EC (left)**x**CHB_ODI (left)**x**time (p=0.03, f=4.58) and tau_RSC (left)**x**CHB_ODI (left)**x**time (p=0.04, f=4.4) for predicting cognitive decline over three years.

**Conclusion:**

Our research has revealed significant connections between the tau accumulation in tau epicenters and posterior regions and the microstructures of neurites along CHB, suggesting that CHB plays a crucial role in tau spreading and cognitive decline.